# Cognitive function and brain structure after recurrent mild traumatic brain injuries in young-to-middle-aged adults

**DOI:** 10.3389/fnhum.2015.00228

**Published:** 2015-05-21

**Authors:** Jonathan List, Stefanie Ott, Martin Bukowski, Robert Lindenberg, Agnes Flöel

**Affiliations:** ^1^Department of Neurology, Charité Universitätsmedizin BerlinBerlin, Germany; ^2^Center for Stroke Research Berlin, Charité Universitätsmedizin BerlinBerlin, Germany; ^3^NeuroCure Cluster of Excellence, Charité Universitätsmedizin BerlinBerlin, Germany

**Keywords:** concussion, cortical thickness, MRI, cognition, traumatic brain injury

## Abstract

Recurrent mild traumatic brain injuries (mTBIs) are regarded as an independent risk factor for developing dementia in later life. We here aimed to evaluate associations between recurrent mTBIs, cognition, and gray matter volume and microstructure as revealed by structural magnetic resonance imaging (MRI) in the chronic phase after mTBIs in young adulthood. We enrolled 20 young-to-middle-aged subjects, who reported two or more sports-related mTBIs, with the last mTBI > 6 months prior to study enrolment (mTBI group), and 21 age-, sex- and education matched controls with no history of mTBI (control group). All participants received comprehensive neuropsychological testing, and high resolution T1-weighted and diffusion tensor MRI in order to assess cortical thickness (CT) and microstructure, hippocampal volume, and ventricle size. Compared to the control group, subjects of the mTBI group presented with lower CT within the right temporal lobe and left insula using an a priori region of interest approach. Higher number of mTBIs was associated with lower CT in bilateral insula, right middle temporal gyrus and right entorhinal area. Our results suggest persistent detrimental effects of recurrent mTBIs on CT already in young-to-middle-aged adults. If additional structural deterioration occurs during aging, subtle neuropsychological decline may progress to clinically overt dementia earlier than in age-matched controls, a hypothesis to be assessed in future prospective trials.

## Introduction

Traumatic brain injury (TBI) is a major cause of acquired disability worldwide in young adults. About 90% of all TBIs are mild TBIs (mTBIs), and >85% of patients report full recovery in clinical and neuropsychological terms within days or weeks after mTBI (Williams et al., 2010). In contrast to subjective resolution of symptoms, several studies indicate that mTBI may not be a benign condition: In the acute and subacute phases (i.e., time period up to 28 days after injury) (Mayer et al., 2010; Shumskaya et al., 2012; Ling et al., 2013), transient cognitive deficits were noted (Maroon et al., 2000; Williams et al., 2010), as well as cortical gray matter abnormalities on diffusion tensor imaging (DTI) (Mayer et al., 2010; Ling et al., 2013). Here increased gray matter fractional anisotropy (FA) most likely reflected cytotoxic edema and subsequent cellular repair mechanisms (Mayer et al., 2010; Ling et al., 2013). Furthermore, long term sequelae in older adults who sustained mTBIs in their youth include depression (Strain et al., 2013), accelerated cognitive decline (Guskiewicz et al., 2005) and dementia (Smith et al., 2013). This indicates that slight but distinct changes in brain architecture may not only persist, but even accentuate as individuals grow older (Moretti et al., 2012). First direct evidence for persistent negative effects in the chronic phase after mTBIs stems from studies of cortical physiology in young adults. Here, it was demonstrated that recurrent mTBIs induce persistent elevations of GABA_B_-mediated intracortical inhibition in primary motor cortex in young athletes, associated with suppressed long-term potentiation (LTP-) and long-term depression (LTD)-like plasticity (De Beaumont et al., 2012), a finding more pronounced in subjects with higher number of mTBIs (De Beaumont et al., 2007). However, these findings are yet unclear, as other studies could not confirm any group differences regarding neurophysiological measurements using TMS and proton MR spectroscopy (Tremblay et al., 2014a). Regarding morphologic changes, there is evidence that juvenile mTBI induces long term degenerative changes (Keightley et al., 2014). With regard to microstructural changes, a recent DTI study reported persistent widespread changes in white matter microstructure in retired athletes with a history of mTBI (Tremblay et al., 2014b).

mTBI occurs frequently in popular sports like soccer, American football, or ice hockey, with approximately 5% of subjects sustaining at least one concussion during the course of a single season (Guskiewicz et al., 2000), suffering from high re-occurrence rates (Guskiewicz et al., 2003), and severe long-term sequelae of recurrent mTBIs including dementia and depression. Therefore, it is of major importance from both a clinical and a socio-economic viewpoint to more closely delineate early detrimental changes after recurrent mTBIs. Particularly, while the neurophysiological sequelae have been described in detail previously (De Beaumont et al., 2012). the impact of recurrent mTBIs on brain structure and function in the chronic phase in young adults are poorly understood.

Focusing on the chronic stage (>6 months after the last injury), we here investigated in a case control study design whether young-to-middle-aged adults after recurrent mTBIs showed alterations in cognitive function, cortical thickness, or gray matter microstructure, as previously described for the subacute phase after single mTBI (Ling et al., 2013). Our main hypothesis was that individuals with a history of recurrent mTBI score lower on cognitive tests and show deteriorations at a brain structural level even in younger adulthood.

## Methods

### Participants

The Study was performed between October 2012 and July 2013. For the mTBI group, 20 subjects (mean age ± SD 25.5 ± 5.3 years, 2 women, 15.3 ± 1.9 years of education, amateur athletes) were recruited from local sport clubs in Berlin and intranet advertisements on the website of the Charité Universitätsmedizin Berlin. For diagnosis of individual mTBI we used a standardized questionnaire. Diagnosis of mTBI required reporting of either confusion for less than 24 h and/or loss of consciousness for less than 30 min following head injury (American Academy of Neurology Practice, 1997). Subjects of the mTBI group had to report sustaining at least 2 mTBIs (2.9 ± 1.5 mTBIs, range 2–7), with the last mTBI taking place at least 6 months prior to study enrolment. A group of 21 healthy subjects (mean age 25.7 ± 5.2 years, 2 women, 15.5 ± 1.9 years of education) without history of (m)TBI was recruited as a control. Healthy controls were recruited from the same local sports clubs (see above) or via intranet advertisements on the website of the Charité.

The study was approved by the local ethics committee of the Charité University Hospital in Berlin/Germany, and performed in accordance with the Declaration of Helsinki. All subjects provided written informed consent, and subsequently underwent neuropsychological testing and MRI.

### Neuropsychological testing

We employed a set of standardized neuropsychological assessments targeting different cognitive domains (Lezak, 2004), in order to detect deficits in domains previously reported as impaired after mTBI (e.g., Matser et al., 1999; Belanger and Vanderploeg, 2005; Monti et al., 2013). Each participant underwent a comprehensive neuropsychological test battery. The German version (Helmstaedter et al., 2001) of the Auditory Verbal Learning Test (AVLT) was used to assess learning ability in the verbal domain (sum of trials 1–5 of word list learning; AVLT, sum 1–5); retrieval from verbal memory was tested by a delayed recall task 30 min later (AVLT 7) to evaluate episodic memory in the verbal domain. Processing speed was assessed with the trail making test (TMT) A and B. For TMT-A, participants were instructed to connect a set of 25 numbers as fast and as accurately as possible, for part B (TMT-B) to alternate between connecting numbers and letters as fast and as accurately as possible. Verbal fluency was assessed with the Regensburger Verbal Fluency Test (RWT), using “S” words and “G/R” words (alternating) for phonemic fluency, “food” and “clothes/flowers” (alternating) for category fluency. For the Rey-Osterrieth Complex Figure Test (ROCF), participants were asked to reproduce a complicated line drawing, first by copying it freehand (recognition), and then drawing from memory (recall). This tasks examined visuospatial skills and episodic memory in the visuospatial domain. Digit span forward and backward (as taken from the (Revised Wechsler Memory Scale) assessed working memory performance. Neuropsychological testing was administered in all but one subject of the mTBI group since he was a non-native German speaker. MRI data (see below) of this subject was included in all MRI analyses. All neuropsychological tests were performed by a medical student who was trained by the lead neuropsychologist of the Neurology Department prior to the study.

### Post-concussive symptoms

Post-concussive symptoms were assessed in the mTBI group by a German version of a standardized questionnaire [Pittsburgh Steelers Post-Concussion Scale (PSPS)] (Maroon et al., 2000). The PSPS aims to detect somatic (balance problems, nausea, headache), neuropsychiatric (irritability, sadness, nervousness, fatigue, trouble falling asleep), and cognitive symptoms (difficulties in concentrating, difficulties in remembering) that may occur after mTBI.

### MRI

#### Image acquisition

MRI were obtained on a 3T Siemens TRIO MR system using a 12-channel head coil at the Berlin Center of Advanced Neuroimaging. We acquired diffusion-weighted images (*TR* = 7500 ms, *TE* = 86 ms, 61 axial slices, voxel size of 2.3 × 2.3 × 2.3 mm^3^; 64 directions with a *b*-value of 1000 s/mm^2^ and 10 b0) and high-resolution T1-weighted MPRAGE images (*TR* = 1900 ms, *TE* = 2.52 ms, 192 sagittal slices, voxel-size of 1 × 1 × 1 mm^3^, flip angle = 9°). In the mTBI group, an additional fluid-attenuated inversion recovery sequence (FLAIR) was acquired.

#### Cortical thickness analyses

The FreeSurfer reconstruction pipeline (http://surfer.nmr.mgh.harvard.edu, version 5.3) was used for CT analyses. The processing of T1-weighted images included removal of non-brain tissue (Segonne et al., 2004), automated Talairach transformation and intensity normalization (Sled et al., 1998), and surface deformation to detect gray matter/white matter and gray matter/cerebrospinal fluid boundaries (Fischl et al., 2001). The resulting representation of CT was calculated as the distance between tissue boundaries (gray matter/white matter and gray matter/cerebrospinal fluid) (Fischl and Dale, 2000). The surface models of each subject were inspected visually for accuracy.

**Figure 1:**
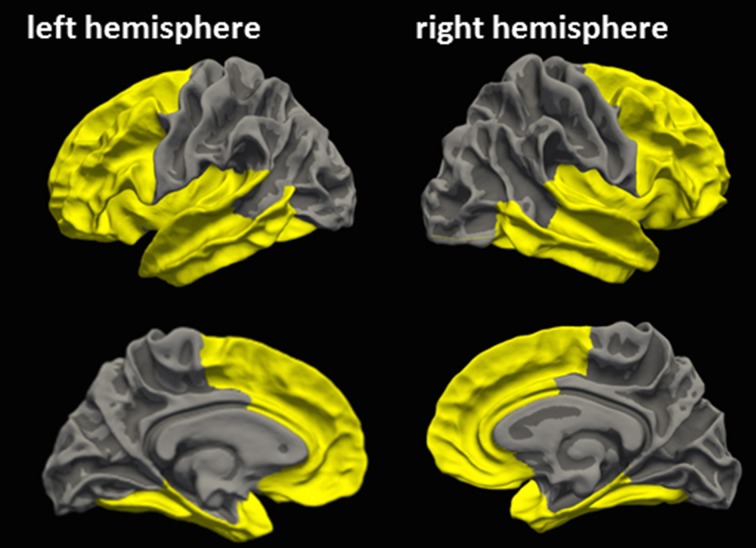
**A priori cortical ROI covering frontotemporal cortical regions most susceptible to mTBI**. Yellow clusters indicate the a priori cortical ROI, which was defined based on FreeSurfer standard labels according to Ling et al. (2013), in order to cover cortical areas most susceptible to mTBI (Bigler and Maxwell, 2012). This frontotemporal ROI comprises the rostral and caudal anterior cingulate area, entorhinal area, insula, superior frontal area, caudal middlefrontal area, pars opercularis, inferior temporal area, lateral orbitofrontal area, medial orbitofrontal area, middle temporal area, pars orbitalis, pars triangularis, rostral middle frontal area, superior temporal area, frontal pole, and temporal pole, fusiform, parahippocampal, transversetemporal of each hemisphere.

Similar to Ling et al. (2013), an a priori cortical ROI was defined based on FreeSurfer standard labels from the Desikan Atlas (Desikan et al., 2006), in order to cover frontotemporal cortical regions most susceptible to mTBI (ROI_FT_, Figure 1) (Bigler and Maxwell, 2012). Mean CT of ROI_FT_ were extracted for each hemisphere in each subject.

For whole-brain cortical thickness analyses, we calculated cortical maps at the vertex-wise level by means of a general linear model (GLM) approach, which is implemented in the graphical user interface QDEC (Query Design Estimate Contrast) from FreeSurfer (https://surfer.nmr.mgh.harvard.edu/fswiki/FsTutorial/QdecGroupAnalysis_freeview). Vertex-wise CT analyses were further employed for each hemisphere over ROI_FT_ with “group” as factor. In case of differences between groups, a second GLM with “number of mTBIs” as factor was applied within the mTBI group, and for exploratory purposes across all subjects. Individual CT maps were registered to the standard template and smoothed with a Gaussian kernel of 20 mm FWHM. Results were corrected for multiple comparisons at *p* < 0.01 using Monte Carlo Simulation on a cluster-wise level (Hagler et al., 2006).

Regarding DTI analyses, ROI_FT_ of the left and right hemisphere were merged using FSL (http://www.fmrib.ox.ac.uk). Fractional anisotropy (FA) and mean diffusivity (MD) of the ROI_FT_ were then extracted for each subject using FreeSurfer. Each subject's diffusion maps (FA and MD separately) were then non-linearly normalized to Talairach space. For both FA maps and MD maps, voxel-based analyses (GLM approach) were run over bilateral ROI_FT_ with “group,” and in case of significant group differences with “number of mTBIs” as factor, corrected for multiple comparisons at *p* < 0.01 using Monte Carlo simulations.

#### Subcortical analyses

For subcortical analyses we used the FreeSurfer subcortical segmentation pipeline (Fischl et al., 2002). To assess general brain atrophy, we extracted lateral ventricle volume as atrophy parameter. Enlargement of lateral ventricles is a common finding in neurodegenerative diseases following shrinkage of brain structures surrounding the ventricles, and is regarded as a sensitive marker for brain atrophy (Nestor et al., 2008; Bigler, 2013). According to previous studies (Monti et al., 2013), Hippocampal and lateral ventricle volumes, as well as gray matter volume were adjusted for individual intracranial volume (ICV), using the automated measure of ICV, provided by FreeSurfer. We used the formula:

adjusted volume=raw volume−bx(ICV−mean ICV)

The coefficient b represents the slope of regression of a region of interest (ROI) volume on ICV. In an exploratory approach, we also calculated volumes of the thalamus and the caudate nucleus, adjusted for individual ICV. Furthermore, we extracted FA and MD from hippocampal ROIs for group wise comparison.

### Statistical analyses

All statistical analyses outside FreeSurfer were carried out using SPSS (Version 22, IBM, Chicago/Illinois). In order to obtain an overview of the performance across different cognitive domains, and to address the issue of specificity of deficits, single-test Z-scores were generated using the mean values and standard deviations of the control subjects (mean score of 0 and standard deviation of 1). We used the formula:

Z=(X−Xmean)/SD

Xmean and SD here refer to the control group.

This approach enabled direct comparison of performance in different cognitive domains. For timed tests, the sign of the Z-score was reversed so that high performance resulted in a higher score in all tests. To determine meaningful composite scores, each cognitive test was assigned to one cognitive domain (**Table 2**). Mean Z-scores of cognitive domains were then calculated by using the mean of the individual test's Z-scores. In a first step, differences between groups in baseline characteristics, neuropsychological test results, cortical thickness and cortical diffusion for whole brain and specific ROIs were assessed with two-tailed two-sample *t*-tests. Group differences in CT analyses were analyzed using FreeSurfer. In case of significant group differences in any of these measurements, Pearson's correlation coefficients were used to probe associations between MRI measurements, cognitive scores, and number of concussions. We provided Bonferroni correction for multiple comparisons in neuropsychological test results. Statistical tests outside FreeSurfer were considered significant at a level of *p* < 0.05.

## Results

### Baseline characteristics

Subjects of the mTBI group and the control group did not differ with regard to age, sex and years of education, all *p*'s > 0.5). Individual characteristics of subjects of the mTBI group are provided in Table 1.

**Table 1:** **Detailed characteristics of the mTBI subjects**.

**Patient no**	**Age**	**Sex**	**Years of education**	**Mechanism of last mTBI**	**No of mTBI**	**AAN-Rating of last mTBI**	**Duration since last mTBI (months)**
1	27	m	17	Ice hockey	2	3	n.a.
2	41	m	15.5	Ice hockey	2	2	6
3	29	m	18	Ice hockey	2	1	13
4	22	f	15.5	Skating	4	3	27
5	21	m	15.5	Rugby	3	3	33
6	26	f	17.5	Fall	5	3	35–36
7	26	m	15.5	Football	3	2	54
8	24	m	17.5	Football	2	2	27–30
9	24	m	15.5	Soccer	3	1	7
10	20	m	15.5	Fall	2	3	14
11	19	m	15.5	Soccer	2	2	9
12	23	m	15	Football	3	2	10
13	21	m	15.5	Football	2	1	22
14	25	m	13	Football	2	1	10–15
15	23	m	10	Football	2	2	12
16	36	m	13	Football	3	1	132
17	24	m	15.5	Football	3	2	11–15
18	25	m	13	Football	6	2	7
19	30	m	18	Mountainbike	7	1	11
20	24	m	15.5	Kickboxing	2	1	32–44

*AAN, American Association of Neurology; mTBI, mild traumatic brain injury*.

### Neuropsychological testing

Subjects of the mTBI group scored trend-wise lower in all tests, as indicated by negative *Z*-values (Table 2 and Supplementary Material), except visuospatial skills which were almost identical. Differences between groups reached significance in verbal fluency tasks (uncorrected), however non-significant after Bonferroni correction (critical *p* < 0.0042). To test whether number of mTBIs was associated with impaired verbal fluency, we performed correlation analyses. Number of mTBIs were not correlated with cognitive scores within the mTBI group (*p* > 0.2).

**Table 2:** **Neuropsychological test results of mTBI subjects and control subjects**.

**Cognitive domain**	**Test**	**mTBI group**	**Control group**	***T***	***p***
Processing speed		−0.34±0.80	0±0.86	−1.242	0.223
	TMT-A	−0.40±1.13 ((35 ± 47))	0±1 (21 ± 7)	−1.135	0.264
	TMT-B	−0.30±0.65 (52 ± 12)	0±1 (46 ± 19)	−1.070	2.929
Verbal memory		−0.22±0.85	0±0.96	−0.763	0.451
	AVLT, sum 1–5	−0.32±0.70 (55 ± 6)	0±1 (58 ± 9)	−1.113	0.273
	AVLT 7	−0.13±1.13 (12 ± 3)	0±1 (12 ± 2)	−0.386	0.701
Working memory		−0.24±0.69	0±0.89	−0.930	0.359
	Digit span, forward	−0.47±0.80 (8 ± 2)	0±1 (9 ± 2)	−1.589	0.121
	Digit span, backward	−0.01±0.85 (8 ± 2)	0±1 (8 ± 2)	−0.046	0.963
Verbal fluency		−0.56±0.48	0±0.78	−2.584	0.014
	RWT S words	−0.60±0.83 (9 ± 4)	0±1 (12 ± 5)	−1.98	0.050
	RWT G/R words	−0.81±0.73 (8 ± 3)	0±1 (11 ± 4)	−2.773	0.009
	RWT food	−0.48±0.89 (14 ± 6)	0±1 (18 ± 6)	−1.543	0.132
	RWT clothes/flowers	−0.35±0.65 (5 ± 3)	0±1 (7 ± 4)	−1.248	0.220
Visuospatial skills		0.13±0.82	0±0.75	0.521	0.606
	Rey figure	0.08±0.74 (36 ± 1)	0±1 (36 ± 1)	0.279	0.782
	Rey recall	0.18±1.13 (27 ± 4)	0±1 (26 ± 4)	0.535	0.596

*Scores reflect mean Z-scores (raw test scores). Groups were compared by unpaired t-tests*.

*mTBI, mild traumatic brain injury; TMT, Trail making test; AVLT, auditory verbal learning test; RWT, Regensburg word fluency task*.

### MRI measurements

Visual inspection of anatomical T1-images in both groups and FLAIR-images in the mTBI group was done by a certified neuroradiologist, No lesions were found.

Normalized gray matter volume, as well as volumes of the thalamus and the caudate nucleus volume were comparable between groups (Table 3). Regarding CT, a whole brain analysis revealed no differences between groups. Within the ROI_FT_, no differences between groups were noted either for mean CT, mean FA, and mean MD. Hippocampal volumes and ventricle sizes were like-wise comparable between groups (all *p* ≥ 0.17, Table 3).

**Table 3:** **Morphometric and diffusion MRI data of mTBI group and control group**.

**Measure-ment**	**ROI**	**mTBI group**	**Control group**	***T***	***p***
CT (mm)	ROI_FT_ CT left	3.350±0.171	3.318±0.188	0.627	0.534
	ROIFT CT right	3.301±0.171	3.358±0.072	−1.399	0.170
Volume (mm^3^)	Grey matter volume	782,958±26,649	782,623±21,916	−0.044	0.965
	Thalamus volume left	8816±443	7968±615	−0.096	0.924
	Thalamus volume right	8166±443	8795±668	−1.176	0.247
	Caudate nucleus volume left	3882±473	3995±399	0.83	0.412
	Caudate nucleus volume right	3708±398	3903±474	1.424	0.163
	Ventricular volume left	6570±4117	7559±4286	−0.753	0.456
	Ventricular volume right	7158±4456	7538±4449	−0.273	0.786
	Hippocampus volume left	4642±403	4568±329	0.638	0.527
	Hippocampus volume right	4536±367	4439±313	0.905	0.371
Fractional anisotropy	ROIFT FA left	0.140±0.007	0.140±0.007	0.039	0.969
	ROIFT FA right	0.142±0.008	0.140±0.008	0.883	0.383
	Hippocampus FA left	0.171±0.0172	0.162±0.0170	1.775	0.084
	Hippocampus FA right	0.175±0.0172	0.164±0.0172	1.920	0.062
Mean diffusivity (×10^−4^)	ROIFT MD left	9.58±0.507	9.62±0.498	−0.252	0.802
	ROIFT MD right	9.37±0.496	9.48±0.512	−0.675	0.504
	Hippocampus MD left	10.47±0.513	10.43±0.507	0.279	0.781
	Hippocampus MD right	10.53±0.513	10.67±0.483	−0.891	0.378

*Groups were compared by unpaired t-tests*.

*mTBI, mildtraumatic brain injury; CT, cortical thickness; ROI, region of interest; FA, fractional anisotropy; MD, mean diffusivity*.

**Figure 2:**
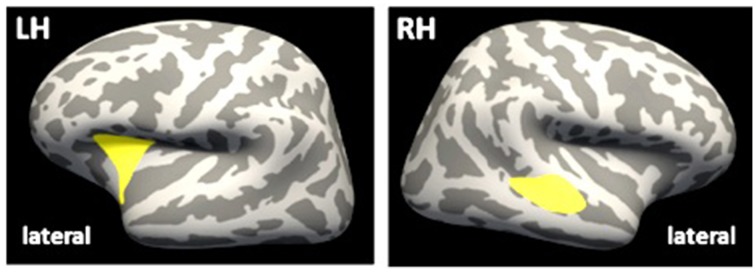
**Differences in CT between groups**. Inflated brain, lateral view. Yellow clusters indicate significant lower CT in mTBI group, as compared to control group. LH, left hemisphere; RH, right hemisphere; CT, cortical thickness.

In a vertex-wise GLM approach within the ROI_FT_, clusters of significant CT differences between groups emerged within the right hemisphere (superior temporal cortex: 2185 vertices, 929 mm^2^, cluster-wise *p* < 0.01; maximum *t* = 4.119, MNI-coordinates: 50.2, −25.7, −2.1; Figure 2) and left hemisphere (insula: 2061 vertices, 753 mm^2^, MNI-coordinates: −29.0, 15.5, 9.5, cluster-wise *p* < 0.01, maximum *t* = 3.376), indicating lower CT in the mTBI group within the left superior temporal cortex and the right insula, when compared to controls. When applying a voxel-wise GLM within the ROI_FT_ on FA or MD, no significant differences between the groups were detected.

#### Associations between number of mTBI and CT

**Figure 3:**
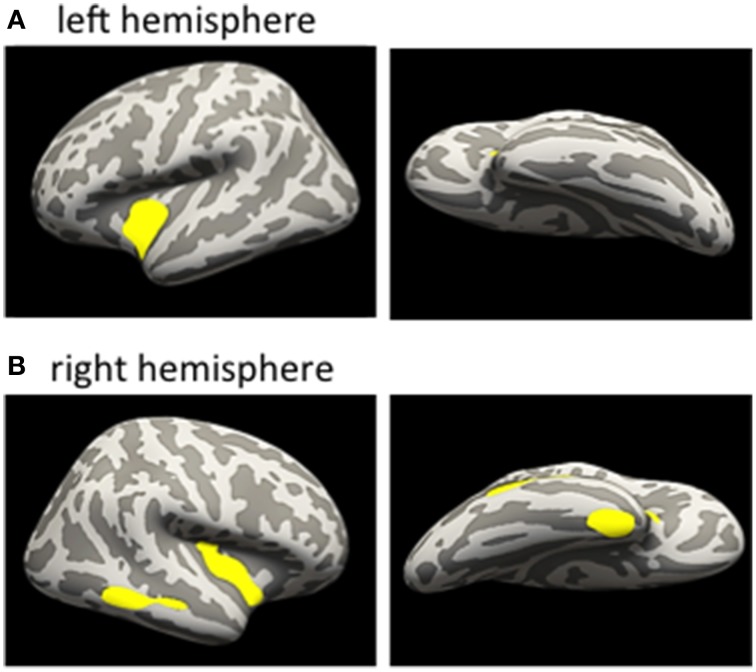
**Correlational analyses between number of mTBI and CT within the mTBI group**. Inflated brain. Clusters reveal negative correlation between number of mTBI and CT; **(A)** left hemisphere, correlation within the mTBI group; **(B)** right hemisphere, correlation within the mTBI group; for details see text. No positive correlations were found. mTBI, mild traumatic brain injury; CT, cortical thickness.

In a first step, we addressed the question whether cortical thinning in the mTBI group was related to the number of mTBIs. We therefore conducted correlation analyses within the significant cluster of lower CT identified in the mTBI group in the between-group analysis. Within the mTBI group, higher number of mTBIs correlated with lower CT of the ROI of the cluster in the left insula (*r* = −0.492, *p* = 0.033) and right superior temporal cortex (*r* = −0.518, *p* = 0.023) (uncorrected). A vertex-wise GLM with “number of mTBIs” as factor within the mTBI group further revealed clusters within the insula bilaterally (insula left: 2038 vertices, 851 mm^3^, MNI-coordinates: −36.1, −4.3, −9.5; insula right: 2575 vertices, 991 mm^3^, MNI-coordinates: 36.6, −11.4, −0.5) and the middle temporal cortex (1015 vertices, 602 mm^3^, MNI-coordinates: 57.5, −45, −8) and fusiform/entorhinal area of the right hemisphere (979 vertices, 575 mm^3^, MNI-coordinates: 35.8, −3.8, −38.7) (Figures 3A,B). Details regarding cluster sizes and sites are provided in Table 4.

**Table 4:** **General linear model results regarding number of mTBI and CT in the mTBI group**.

**Cluster**	**Size (mm^2^)**	**# Vertices**	**Maximum *t***	***X***	***Y***	***Z***
Insula cortex, left	851	2038	−5.287	−36.1	−4.3	−9.5
Middle temporal cortex, right	602	1015	−3.885	57.5	−45	−8
Fusiform/entorhinal cortex right	575	979	−3.085	35.8	−3.8	−38.7
Insula cortex, right	991	2575	−2.958	36.6	−11.4	−0.5

*mm, millimeters; X, Y, Z, coordinates in Montreal Neurological Institute space*.

## Discussion

Our data suggest that recurrent mTBIs exerts detrimental effects on cognitive function and cortical thickness in the chronic phase in young-to-middle-aged adults. Specifically, cognitive testing revealed small but consistent deteriorations in cognitive scores, most pronounced for verbal fluency, in the chronic phase after mTBIs.. Moreover, we found that subjects with recurrent mTBIs showed dose-dependent cortical thinning within right temporal lobe and bilateral insula, as compared to subjects without history of mTBI.

In recent years, a number of studies have evaluated cognitive and MRI-based sequelae of mTBI in the acute and subacute phase after mTBI. For example, alterations of white (Mayer et al., 2010) and gray matter microstructure (Ling et al., 2013) have been reported, as well as differences in resting state functional connectivity within the default mode network (Mayer et al., 2011) and motor striatal network (Shumskaya et al., 2012). Other studies focused on long term sequelae of mTBI in older age. Here changes of brain structure and function were even more pronounced than acutely or subacutely after the injury (Monti et al., 2013; Strain et al., 2013), indicating that cognitive and psychiatric deficits after mTBI may not only persist but even accentuate over longer time periods (Moretti et al., 2012). In fact, previous evidence suggest that history of mTBI is a strong risk factor for the development of Alzheimer's dementia (AD) in later life (Mielke et al., 2014), an and earlier onset of AD in subjects who suffered recurrent mTBIs (Guskiewicz et al., 2003). This may in part be due to accelerated neurodegeneration after mTBI, e.g., due to induction of neurotoxic cascades, inflammatory processes (Geddes et al., 1999; Zetterberg et al., 2013), and deposits of hyperphosphorylated tau (Johnson et al., 2010; McKee et al., 2013).

So far, little is known about the first years following the subacute phase after recurrent mTBI in younger subjects. Here, development of overt dementia is unlikely; yet detrimental sequelae like neurotoxicity and inflammation may be set in motion at this time, rendering a more detailed understanding of the changes occurring during this time particularly crucial with regard to prevention of dementia at a later stage.

The present study assessed whether changes in brain structure and gray matter microstructural integrity would be detectable in the chronic phase after recurrent mTBIs in young-to-middle-aged adults. We could demonstrate that subjects with recurrent mTBIs showed significant clusters of cortical thinning, as compared to controls, in right temporal lobe and in left insular cortex. The temporal area identified in this study is one of the first regions showing cortical thinning in the precursor of AD, mild cognitive impairment (Fjell et al., 2014). Moreover, lower CT in the temporal area was associated with lower verbal fluency, known to be among the first cognitive functions to deteriorate after TBI (Belanger and Vanderploeg, 2005; Williams et al., 2010) and in AD (Albert, 2008). The insular cortex plays an important role in emotional processing (Craig, 2009). Thus, alterations in the chronic phase after recurrent mTBIs in younger subjects may render them more prone to develop depression in later life (Stern et al., 2013), as has been described in retired athletes with history of mTBI (Strain et al., 2013).

Moreover, we were able to demonstrate that higher numbers of mTBIs were associated with lower cortical thickness in right entorhinal area. The entorhinal area is known to show volume loss already in the early course of AD, and correlates significantly with the severity of the disease (Juottonen et al., 1998), Our finding of a dose-dependent effect of mTBIs onto cortical thickness of entorhinal area thus supports induction of neurodegenerative changes as a function of recurrent mTBIs. This is further corroborated by previous findings in older subjects with history of recurrent mTBIs that demonstrated higher prevalence of cognitive deficits after 3 or more mTBIs compared to 1 or 2 mTBI (Guskiewicz et al., 2005).

Our current results support the hypothesis that recurrent mTBIs induce changes in cortex morphology that may deteriorate over years, although subjects may not suffer from clinically overt post-concussive symptoms directly after mTBI, and may not report cognitive deficits in the first years after the injuries. We therefore hypothesize that recurrent mTBIs may induce distinct and persistent alterations in gray matter structure, similar to what has been reported in brain physiology patterns (De Beaumont et al., 2007, 2012). In contrast, previously reported cortical DTI alterations from the acute phase after mTBI (Ling et al., 2013) may normalize in the chronic phase. Cortical thinning is known to be one of the most sensitive non-invasive markers of incipient neurodegeneration, as reported in previous studies in AD (Dickerson et al., 2009) and mTBI (Tremblay et al., 2012), indicating neuronal shrinkage and reduction in arborization along with a loss of myelin sheaths, and possibly loss of neuronal, glial, or other important cellular components such as neuropil volume (Dickerson et al., 2009). These first subtle changes may then translate into more overt changes in hippocampal and ventricular volume in subjects with recurrent mTBIs at older age (Monti et al., 2013).

In sum, our results suggest subtle abnormalities in cognitive function and brain morphology after recurrent mTBIs even in young-to-middle-aged subjects, most pronounced in individuals with highest number of mTBIs, supporting the dose-dependent effect of mTBIs with regard to neuronal sequelae previously demonstrated in older age (Guskiewicz et al., 2005).

## Limitations

This cross-sectional study has several limitations. First, the number of subjects is small, and results should be replicated in larger samples in the future. Second, differences between groups for cortical thinning were found only in a ROI, and not in a whole brain analysis. Third, the person conducting the neuropsychological tests was not blinded regarding group assignment. However, analysis of MRI scans was conducted with blinding regarding group assignment. Fourth, the number of mTBIs was self-reported in most cases. However, we used standardized and validated questionnaires, similar to previous landmark studies in the field (Guskiewicz et al., 2005; Singh et al., 2014). Fifth, we only investigated brain structure and cognitive performance in the chronic phase after recurrent mTBIs without accounting for the exact time of the last mTBI. In the future, a prospective design including cognitive testing and MRI from the acute over subacute into chronic phase after each mTBI will help determine the time-course of cognitive and brain structural changes after injuries.

## Conclusion

This study provides further insight into chronic effects of recurrent mTBIs on cognitive functions and gray matter structure, possibly mediated by TBI-induced neurotoxic, inflammatory, or immunomodulatory processes (Walker and Tesco, 2013; Lucke-Wold et al., 2014). In older age, these first subtle alterations may promote clinically apparent cognitive decline and dementia, a hypothesis to be evaluated by longitudinal assessments of young-to-middle-aged subjects with recurrent mTBIs until older age. The present results indicate that CT assessments of specific cortical regions, including the entorhinal area, may be useful as biomarker in order to predict risk of cognitive decline in subjects with recurrent mTBIs. More detailed evaluations of mechanisms that may underlie these changes, including blood- or cerebrospinal fluid-derived parameters (Zetterberg et al., 2013; Shahim et al., 2014) may allow us to develop additional strategies to counteract further neurodegeneration, for example by using immunmodulatory therapies if immune responses were to be implicated in the process (Rodgers et al., 2012). Given the high prevalence of mTBI (Guskiewicz et al., 2000) in an aging society with increasing prevalence of aging-associated diseases like AD (Pressley et al., 2003), understanding the mechanisms of functional and structural decline following recurrent mTBIs is of major medical and socio-economic importance.

## Author contributions

JL: study concept and design, acquisition of data, analysis and interpretation of data, study supervision, writing the manuscript SO: acquisition of data MB: acquisition of data RL: Study concept and design, acquisition of data, critical revision of the manuscript for intellectual content AF: Study concept and design, study supervision, writing the manuscript.

### Conflict of interest statement

The authors declare that the research was conducted in the absence of any commercial or financial relationships that could be construed as a potential conflict of interest.
