# Supplementary material for: Cognitive function and brain structure after recurrent mild traumatic brain injuries in young-to-middle-aged adults
Source: Front Hum Neurosci. 2015 May 21;9:228. doi: 10.3389/fnhum.2015.00228 (PMC4440350; doi:10.3389/fnhum.2015.00228)
Supplement: Supplementary file 1 [file DataSheet1.DOCX]

**SUPPLEMENTARY METHODS**

**Supplementary Figure 1:**

**
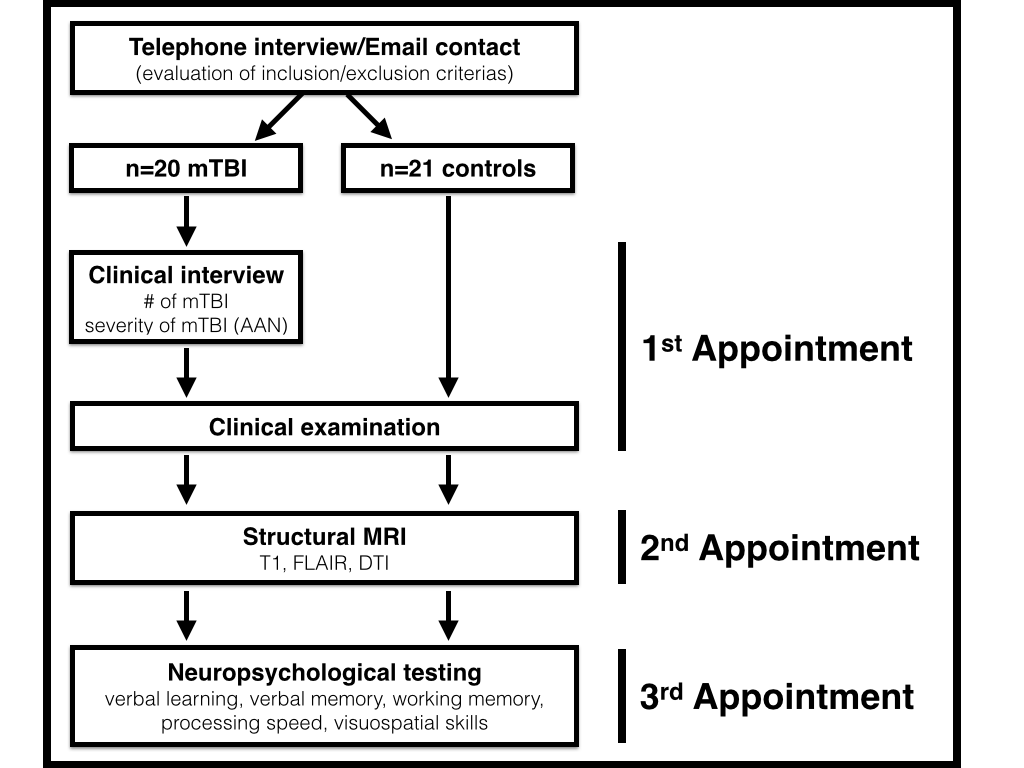
**

**Supplementary Figure 1**: Study outline. After telephone or Email screening for inclusion and exclusion criterias, subjects of the mTBI group underwent a clinical interview (valuation of the number of mTBI, severity of mTBI). Both groups then underwent a clinical examination, structural MRI and neuropsychological testing in three separate sessions. mTBI=mild traumatic brain injury, MRI=magnetic resonance imaging, FLAIR= fluid-attenuated inversion recovery sequence, DTI=diffusion tensor imaging

**Supplementary Figure 2:**


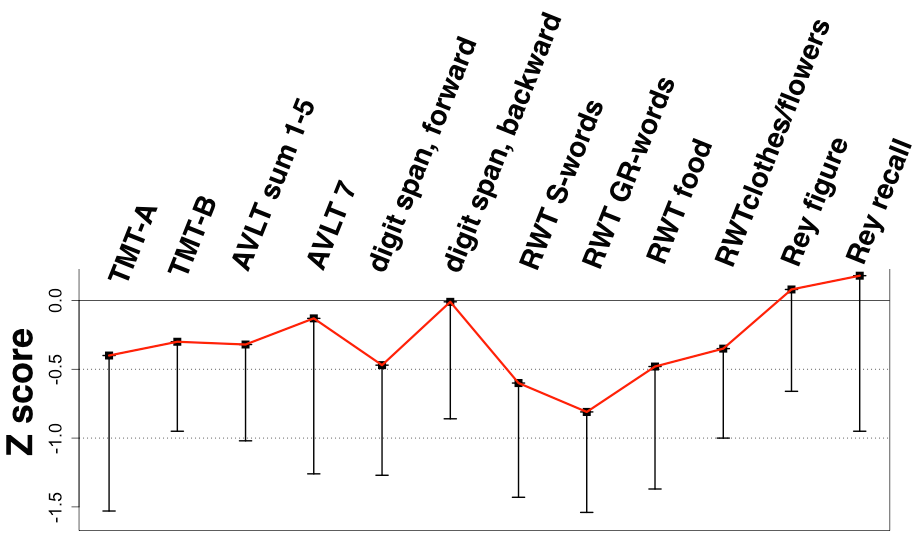


**Supplementary Figure 2:** Neuropsychological performance profile of mTBI subjects. Scores reflect mean Z scores. Mean Z scores ([X-Xmean]/SD) were calculated by using the mean values and standard deviations of the control group (a Z score of 1 corresponds to one standard deviation from test scores in the control group). TMT=trail making test, AVLT=auditory verbal learning test, RWT=Regensburger Wortflüssigkeitstest
